# A Molecular Prognostic Model Predicts Esophageal Squamous Cell Carcinoma Prognosis

**DOI:** 10.1371/journal.pone.0106007

**Published:** 2014-08-25

**Authors:** Hui-Hui Cao, Chun-Peng Zheng, Shao-Hong Wang, Jian-Yi Wu, Jin-Hui Shen, Xiu-E Xu, Jun-Hui Fu, Zhi-Yong Wu, En-Min Li, Li-Yan Xu

**Affiliations:** 1 The Key Laboratory of Molecular Biology for High Cancer Incidence Coastal Chaoshan Area, Shantou University Medical College, Shantou, Guangdong, China; 2 Institute of Oncologic Pathology, Shantou University Medical College, Shantou, Guangdong, China; 3 Department of Biochemistry and Molecular Biology, Shantou University Medical College, Shantou, Guangdong, China; 4 Departments of Oncology Surgery, Shantou Central Hospital, Affiliated Shantou Hospital of Sun Yat-sen University, Shantou, Guangdong, China; 5 Departments of Pathology, Shantou Central Hospital, Affiliated Shantou Hospital of Sun Yat-sen University, Shantou, Guangdong, China; Deutsches Krebsforschungszentrum, Germany

## Abstract

**Background:**

Esophageal squamous cell carcinoma (ESCC) has the highest mortality rates in China. The 5-year survival rate of ESCC remains dismal despite improvements in treatments such as surgical resection and adjuvant chemoradiation, and current clinical staging approaches are limited in their ability to effectively stratify patients for treatment options. The aim of the present study, therefore, was to develop an immunohistochemistry-based prognostic model to improve clinical risk assessment for patients with ESCC.

**Methods:**

We developed a molecular prognostic model based on the combined expression of axis of epidermal growth factor receptor (EGFR), phosphorylated Specificity protein 1 (p-Sp1), and Fascin proteins. The presence of this prognostic model and associated clinical outcomes were analyzed for 130 formalin-fixed, paraffin-embedded esophageal curative resection specimens (generation dataset) and validated using an independent cohort of 185 specimens (validation dataset).

**Results:**

The expression of these three genes at the protein level was used to build a molecular prognostic model that was highly predictive of ESCC survival in both generation and validation datasets (*P* = 0.001). Regression analysis showed that this molecular prognostic model was strongly and independently predictive of overall survival (hazard ratio = 2.358 [95% CI, 1.391–3.996], *P* = 0.001 in generation dataset; hazard ratio = 1.990 [95% CI, 1.256–3.154], *P* = 0.003 in validation dataset). Furthermore, the predictive ability of these 3 biomarkers in combination was more robust than that of each individual biomarker.

**Conclusions:**

This technically simple immunohistochemistry-based molecular model accurately predicts ESCC patient survival and thus could serve as a complement to current clinical risk stratification approaches.

## Introduction

Among all types of cancer, esophageal cancer (EC) has the eighth and sixth highest incidence and mortality rates worldwide, respectively [1]. Although esophageal adenocarcinoma (EAC) has become the predominant histological subtype in some western countries, esophageal squamous cell carcinoma (ESCC) remains dominant in China, with almost 90% of newly diagnosed patients exhibiting this cancer subtype [2]. The 5-year survival rate for ESCC remains dismal, despite improvements in treatments such as surgical resection and adjuvant chemoradiation. In current clinical practice, pathological tumor-node-metastasis (pTNM) stage is considered the optimal prognostic indicator. However, this clinical staging approach is limited in its ability to precisely stratify patients for treatment options due to wide variation in survival rates, such as that observed among T3N1 patients [3]. Clearly, identifying effective biomarkers to complement current clinical staging approaches is highly important. According to national guidelines [4], [5], biomarkers should be sensitive, specific, cost-effective, fast, robust against variability, and more accurate than current clinical stages. A single biomarker, however, may be unlikely to fulfill all of these requirements.

In recent decades, the identification of combinations of biomarkers instead of single biomarkers has become a popular research endeavor. Multi-gene signatures of breast cancer, colorectal cancer, esophageal and gastroesophageal junction adenocarcinoma, and other cancer types have served as successful prognostic indicators [3], [6]–[9]. The ability of these gene signatures to accurately predict survival provides a foundation on which to build molecular classification systems and individualized treatment approaches. To date, however, the application of molecular prognostic signatures is less advanced for ESCC than for other cancer subtypes.

In a previous study, we showed that ESCC was associated with the overexpression of Fascin, which was regulated by phosphorylated Specificity protein 1 (p-Sp1) via activation of the epidermal growth factor (EGF)/extracellular signal-regulated kinase (ERK) signaling pathway [10]. Although the clinical significance of this pathway remains unclear, the EGF receptor (EGFR), a transmembrane glycoprotein belonging to the HER family of receptors, is recognized as a negative prognostic indicator [11], [12] and has shown clinical relevance as a molecular target of cancer therapies [13], [14]. Fascin, an actin bundling protein, is also recognized as a prognostic indicator, with its overexpression associated with aggressive clinical phenotypes and poor survival [15]–[17]. Based on the clinical significance of EGFR and Fascin, we hypothesized that a combination of molecules from the EGFR/ERK/Fascin signaling pathway could accurately predict cancer outcome. Indeed, we found that a three-gene signature comprised of expression of EGFR, p-Sp1, and Fascin proteins independently predicted ESCC patient survival. This molecular prognostic model could give rise to a new molecular stratification system and provide a useful framework for future work on prognostic signatures for ESCC and other cancers.

## Materials and Methods

### Patients and specimens

Paraffin-embedded tissues were derived from two independent cohorts of ESCC patients undergoing curative resection at Shantou Central Hospital between 2007 and 2009 (generation dataset, n = 130) or between 1987 and 1997 (validation dataset, n = 185). Patients in the generation dataset were followed up for a median time period of 35.0 months, with follow-ups terminated on November 9, 2012. Patients in the validation dataset were followed up for a median and maximum time period of 33.6 and 131.3 months, respectively. Overall survival rate (OS) was calculated during the period between surgery and death or final observation. Information on patient age, gender, stage of disease, therapy, and histopathology was obtained from medical records (Table 1). The study was approved by the ethical committee of the Central Hospital of Shantou City and the ethical committee of the Medical College of Shantou University, and written informed consent was obtained from all surgical patients to use resected samples for research.

**Table 1:** The clinicopathological characteristics of two datasets of patients with ESCC.

Clinical and pathological indexes	Generation dataset		Validation dataset	
	No.	%	No.	%
Specimens	130		185	
Mean age	59		58	
Age (year)				
< Mean age	70	53.8	87	47.0
≥ Mean age	60	46.2	98	53.0
Gender				
Male	103	79.2	140	75.7
Female	27	20.8	45	24.3
Differentiation				
G1	21	16.2	44	23.8
G2	97	74.6	111	60.0
G3	12	9.2	30	16.2
T-stage				
T1+T2	17	13.1	32	17.3
T3+T4	113	86.9	153	82.7
N-stage				
N0	63	48.5	122	65.9
N1	67	51.5	63	34.1
M-stage				
M0	130	100	178	96.2
M1	0	0	7	3.8
pTNM-stage				
IA+IB+IIA+IIB	68	52.3	125	67.6
IIIA+IIIB+IIIC+IV	62	47.7	60	32.4
Therapy				
Only Surgery	84	64.6	119	64.3
Surgery + chemo	19	14.6	39	21.1
Surgery + radio	25	19.2	20	10.8
Surgery + chemo + radio*	2	1.6	7	3.8

*, chemo, chemotherapy; radio, radiotherapy.

### Tissue microarrays (TMAs) and immunohistochemistry (IHC)

TMAs were constructed as previously described [17]–[19]. The primary antibodies used in this study were mouse anti-EGFR (ready-to-use; ZSGB-BIO, Beijing, China), rabbit anti-Sp1(phospho T453, 1∶100 dilution; Abcam, Cambridge, UK), and mouse anti-human Fascin-1(clone 55K-2, 1∶100 dilution; Dako, Carpinteria, CA). IHC was carried out using a two-step protocol (PV-9000 Polymer Detection System, ZSGB-BIO, Beijing, China) as previously described [19].

### Evaluation of IHC variables

Tissue sections were independently and blindly assessed by three histopathologists (Cao HH, Wang SH, and Shen JH). Discrepancies were resolved by consensus. The EGFR expression was scored using the HercepTest criterion [20]. EGFR scoring criteria: 0 corresponded to no staining at all, or membrane staining in less than 10% of the tumour cells was observed, 1+ corresponded to a faint/barely perceptible membrane staining was detected in more than 10% of the tumour cells. The cells were only stained in part of their membrane, 2+ corresponded to a weak to moderate staining of the entire membrane was observed in more than 10% of the tumour cells and 3+ was a strong staining of the entire membrane was observed in more than 10% of the tumour cells. EGFR staining was predominantly located in the cell membrane, cytoplasmic staining was considered non-specific and not included in the scoring. For statistical analysis, we divided EGFR scores into two groups; scores of 0–2+ were considered low-expression and scores of 3+ were considered high-expression.

Fascin expression was assessed by staining of cell cytoplasm. Its expression was scored as described by Zhao et al.^17^ Each separate tissue core was scored on the basis of the intensity and area of positive staining. The intensity of positive staining was scored as follows: 0, negative; 1, weak staining; 2, moderate staining; 3, strong staining. The rate of positive cells was scored on a 0–4 scale as follows: 0, 0–5%; 1, 6–25%; 2, 26–50%; 3, 51–75%; 4, >75%. If the positive staining was homogeneous, a final score was achieved by multiplication of the two scores, producing a total range of 0–12. When the staining was heterogeneous, we scored it as follows: each component was scored independently and summed for the results. For example, a specimen containing 25% tumor cells with moderate intensity (1×2 = 2), 25% tumor cells with weak intensity (1×1 = 1), and 50% tumor cells without immunoreactivity (2×0 = 0), received a final score of 2+1+0 = 3. For statistical analysis, we divided Fascin scores into two groups; scores of 0–10 were considered low-expression and scores of more than 10 were considerd high-expression.

p-Sp1 expression was assessed by staining of cell nuclei. Cytoplasmic staining was considered non-specific and not included in the scoring. p-Sp1 expression levels were scored on a scale ranging from 0 to 3+: 0 indicated no positive staining; 1+ indicated only a few scattered stained cells or weak staining in less than 30% of cells within a visual field; 2+ indicated cluster(s) of moderate to strong staining in less than 30% of cells or weak staining in more than 30% of cells; 3+ indicated cluster(s) of moderate to strong staining in more than 30% of cells. For statistical analysis, we divided p-Sp1 scores into two groups; scores of 0–2+ were considered low-expression, and scores of 3+ were considered high-expression.

### Construction of a weighted OS predictive model

Cox proportional hazards regression analysis was used to evaluate the association between biomarker expression and OS. We then constructed a model to estimate risk by summing the expression level of each biomarker (high-expression = 1, low-expression = 0) multiplied by its regression coefficient [21]–[23]. Patients were dichotomized into high- or low-risk groups using the 50th percentile (i.e., median) risk score as a cut-off value.

### Statistical analysis

Statistical analyses were performed using SPSS 13.0 for Windows. Cumulative survival time was calculated by the Kaplan-Meier method and analysed by the log-rank test. Spearman’s two-sided rank correlation was used to explore the correlation levels between three proteins expression. Univariate and multivariate analyses were based on the Cox proportional hazards regression model. Receiver operating characteristic (ROC) curve analysis was used to determine the predictive value of the parameters, and the differences in the area under the curve (AUC) were detected by using GraphPad Prism 5. The Kendall tau-b rank correlation analysis was used to evaluate the association between the expression of the prognostic model and clinicopathological factors. *P* value less than 0.05 was considered statistically significant.

## Results

### IHC characteristics of EGFR, p-Sp1, and Fascin biomarkers

**Figure 1:**
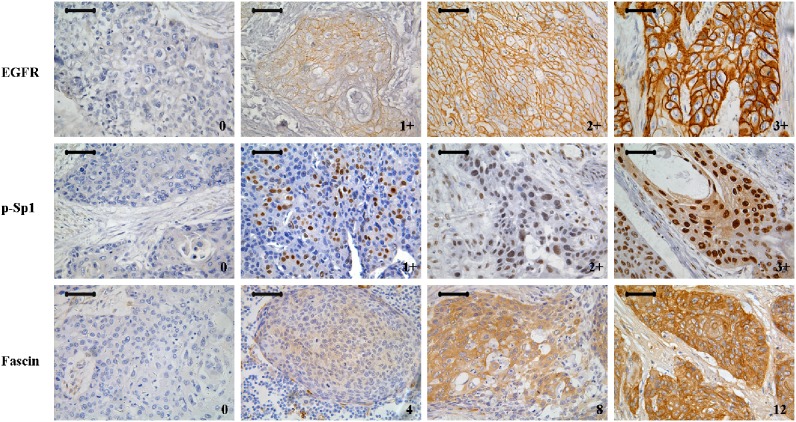
Representative images of IHC staining scores for EGFR, p-Sp1, and Fascin in esophageal squamous cell carcinoma (ESCC). Scale bars = 50 µm.

Three potential biomarkers from the EGFR/ERK/Fascin signaling pathway were stained using IHC. EGFR and p-Sp1 staining were mainly observed in cell membranes and nuclei, respectively, whereas Fascin staining was more diffuse throughout the cytoplasm. Representative images of different staining scores are shown in Figure 1. However, positive staining of EGFR and Fascin was apparent only in basal layer of epithelium tissue adjacent to carcinoma, while p-Sp1 was weak staining in higher granular layer of the epithelium (Figure S1). Our results were the same as other reports in ESCC, while no report of Sp1 in ESCC [24], [25].

### Correlations between the three biomarkers

In both the generation dataset and the validation dataset, the Spearman’s rank correlation showed that the expression of EGFR was closely associated with the Fascin expression (*r* = 0.299, *P* = 0.001 and *r* = 0.154, *P* = 0.037), while no correlation between EGFR and p-Sp1 or between p-Sp1 and Fascin. Detail information was in Figure S2.

### Prognostic significance of EGFR, p-Sp1, and Fascin expression and other clinical/pathological characteristics

In the generation dataset, the 1- and 3-year OS were 83.1% and 57.5%, respectively. In the validation dataset, the 1-, 3-, and 5-year OS were 93.5%, 62.4%, and 50%, respectively. Univariate analysis revealed that the three biomarkers (EGFR, p-Sp1, and Fascin), as well as four pathological factors (Differentiation [G3 vs. G1], N-stage, M-stage, and pTNM-stage), were significantly associated with OS (Table 2). However, EGFR did not significantly predict OS in the generation dataset, perhaps due to heterogeneity in EGFR expression patterns between the two datasets. Kaplan-Meier analysis provided further support that EGFR, p-Sp1, and Fascin were significant predictors of OS in both generation and validation datasets, except for EGFR in the validation dataset (Figure S3). In the generation dataset, the 3-year OS was significantly lower for the p-Sp1 and Fascin high-expression groups than the low-expression groups. In the validation dataset, the 3- and 5-year OS were significantly lower for the EGFR, p-Sp1, and Fascin high-expression groups than the low-expression groups.

**Table 2:** Univariate analyses and Multivariate analysis of factors associated with overall survival.

	Generation dataset				Validation dataset			
	Sig.*	HR	95% CI for HR		Sig.*	HR	95% CI for HR	
Variables			Lower	Upper			Lower	Upper
Univariate analyses								
Age (≥ Mean age vs < Mean age)	0.749	1.084	0.661	1.778	0.711	1.087	0.699	1.692
Gender (Female vs Male)	0.372	1.333	0.709	2.509	0.333	0.762	0.440	1.320
Differentiation	0.095				0.074			
G2 vs G1	0.127	1.853	0.838	4.097	0.163	1.529	0.842	2.778
G3 vs G1	0.030	3.196	1.118	9.137	0.023	2.256	1.121	4.541
T-stage (T3+T4 vs T1+T2)	0.771	0.901	0.444	1.825	0.799	0.928	0.520	1.654
N-stage (N1 vs N0)	0.003	2.174	1.297	3.645	0.000	2.306	1.481	3.593
M-stage^a^ (M1 vs M0)					0.015	2.829	1.228	6.518
pTNM-stage (III+IV vs I+II)	0.002	2.220	1.335	3.690	0.003	1.982	1.270	3.092
Therapy (Comprehensive Therapy^b^ vs Only Surgery)	0.655	0.887	0.525	1.501	0.103	1.443	0.923	2.258
EGFR (high vs low)	0.580	1.151	0.699	1.896	0.038	1.614	1.027	2.536
p-Sp1 (high vs low)	0.004	2.087	1.271	3.425	0.030	1.672	1.052	2.657
Fascin (high vs low)	0.027	1.749	1.065	2.873	0.017	1.721	1.104	2.684
prognostic model (high vs low)	0.001	2.381	1.408	4.029	0.001	2.131	1.348	3.369
Multivariate analysis								
pTNM-tage (III+IV vs I+II)	0.003	2.199	1.319	3.667	0.008	1.826	1.167	2.856
prognostic model (high vs low)	0.001	2.358	1.391	3.996	0.003	1.990	1.256	3.154

*Multivariate analysis, Cox proportional hazards regression model. Variables were adopted for their prognostic significance by univariate analysis.

a , no data because that patients suffering metastasis (M1) were considered inappropriate for curative resection.

b , Comprehensive Therapy including Surgery + chemotherapy, Surgery + radiotherapy and Surgery + chemotherapy + radiotherapy.

### Predictive molecular prognostic model

Our molecular prognostic model was Calculated as Y = (β_1_)×(EGFR)+(β_2_)×(p-Sp1)+(β_3_)×(Fascin), with Y equal to risk score and β_n_ equal to each gene’s coefficient value from univariate Cox proportional hazards regression analysis. In the generation dataset, β_1_ = 0.141, β_2_ = 0.736, and β_3_ = 0.559. In the validation dataset, β_1_ = 0.479, β_2_ = 0.514, and β_3_ = 0.543. Patients were ranked and divided into high- and low-risk groups using the 50th percentile (i.e., median) risk score as the cut-off value.

**Figure 2:**
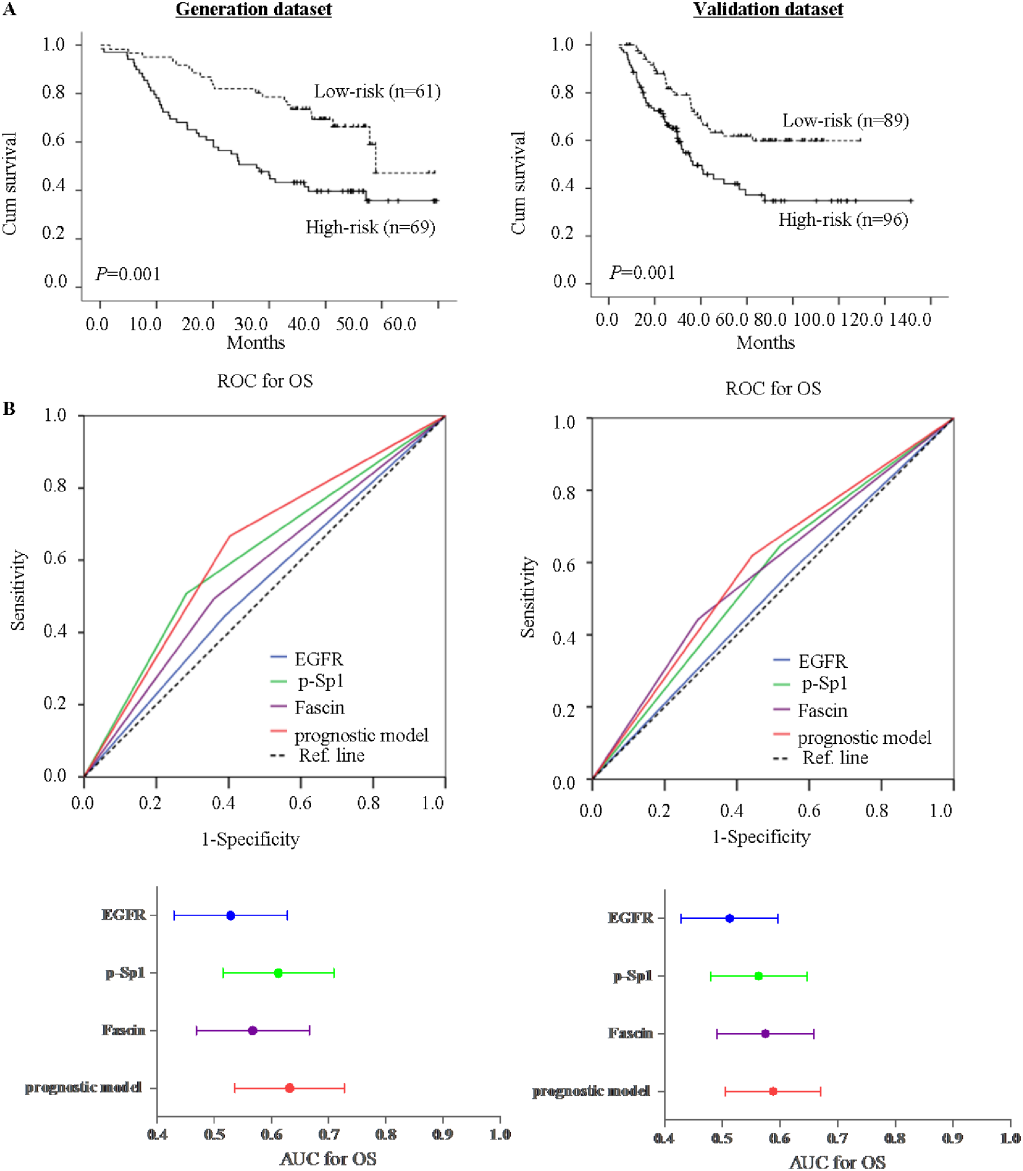
Predictive ability of the molecular prognostic model. A, Kaplan-Meier analysis of OS for low-risk and high-risk ESCC patients based on expression of the molecular prognostic model in generation and validation datasets. B, Predictive ability of the molecular prognostic model compared with individual biomarker shown by receiver operating characteristic (ROC) curves and area under the curve (AUC) in generation and validation datasets.

In the generation dataset, the 3-year OS for the high-risk group was significantly lower than that for the low-risk group (73.6% vs. 43.3%; Figure 2A). Similar results were found in the validation dataset, that the 3- and 5-year OS for the high-risk group were significantly lower than those for the low-risk group (73.6% and 61.8% vs. 51.4% and 37.2%, respectively; Figure 2A). Multivariate Cox proportional hazards regression analysis showed that the three-gene signature, along with pTNM-stage, was a strong and independent predictor of OS (Table 2).

### Predictive power of the molecular prognostic model

In both the generation and validation datasets, receiver operating characteristic (ROC) analysis showed that the predictive power of the prognostic model was higher than that for each biomarker individually. In the generation dataset, specificity and sensitivity were 66.7% and 59.7%, respectively, and area under the curve (AUC) for OS with 95% CI was 0.632. Similar results were found in the validation dataset, with 62% specificity, 55.7% sensitivity, and 0.588 AUC (Figure 2B). Furthermore, in the generation dataset, the predictive ability of the prognostic model was not only higher than that of EGFR, p-Sp1, and Fascin individually but also higher than all clinical/pathological characteristics. However, in the validation dataset, the AUC for the prognostic model was not larger than that for N-stage and pTNM-stage, but specificity and sensitivity were optimal (Figure S4).

### Correlations between the prognostic model and clinical/pathological characteristics

Kendall tau-b correlation analysis indicated that the prognostic model was significantly related to N-stage (Table 3). In the generation dataset, the proportion of high-risk in patients suffering regional lymph node metastasis (N1) were significantly higher than that of high-risk in patients without regional lymph node metastasis (N0) (62.7% [42/67] vs. 42.9% [27/63], *P* = 0.035). Similar results were obtained in the validation dataset (63.5%[40/63] vs. 45.9% [56/122], *P* = 0.030). Other clinical/pathological characteristics such as age, gender, differentiation, T-stage, M-stage, pTNM-stage, and therapy, however, were not significantly different between high-risk and low-risk patients.

**Table 3:** The correlation between molecular prognostic model and clinicopathological characteristics in ESCC.

Variables	Generation dataset		*P* *	Validation dataset		*P* *
	Low-risk	High-risk		Low-risk	High-risk	
Age (year)						
< Mean age	35	35	0.484	41	46	0.883
≥ Mean age	26	34		48	50	
Gender						
Male	44	59	0.083	68	72	0.865
Female	17	10		21	24	
Differentiation						
G1	9	12	0.465	23	21	0.905
G2	45	52		49	62	
G3	7	5		17	13	
T-stage						
T1+T2	4	13	0.066	15	17	1.000
T3+T4	57	56		74	79	
N-stage						
N0	36	27	0.035	66	56	0.030
N1	25	42		23	40	
M-stage						
M0	61	69	-	86	92	1.000
M1	0	0		3	4	
pTNM-stage						
IA+IB+IIA+IIB	37	31	0.081	65	60	0.157
IIIA+IIIIB+IIIC+IV	24	38		24	36	
Therapy						
Only Surgery	38	46	0.714	57	62	1.000
Comprehensive Therapy^a^	23	23		32	34	

*The Kendall’s tall-b test;

a , Comprehensive Therapy including Surgery + chemotherapy, Surgery + radiotherapy and Surgery + chemotherapy + radiotherapy.

### Combination of the prognostic model and N-stage

**Figure 3:**
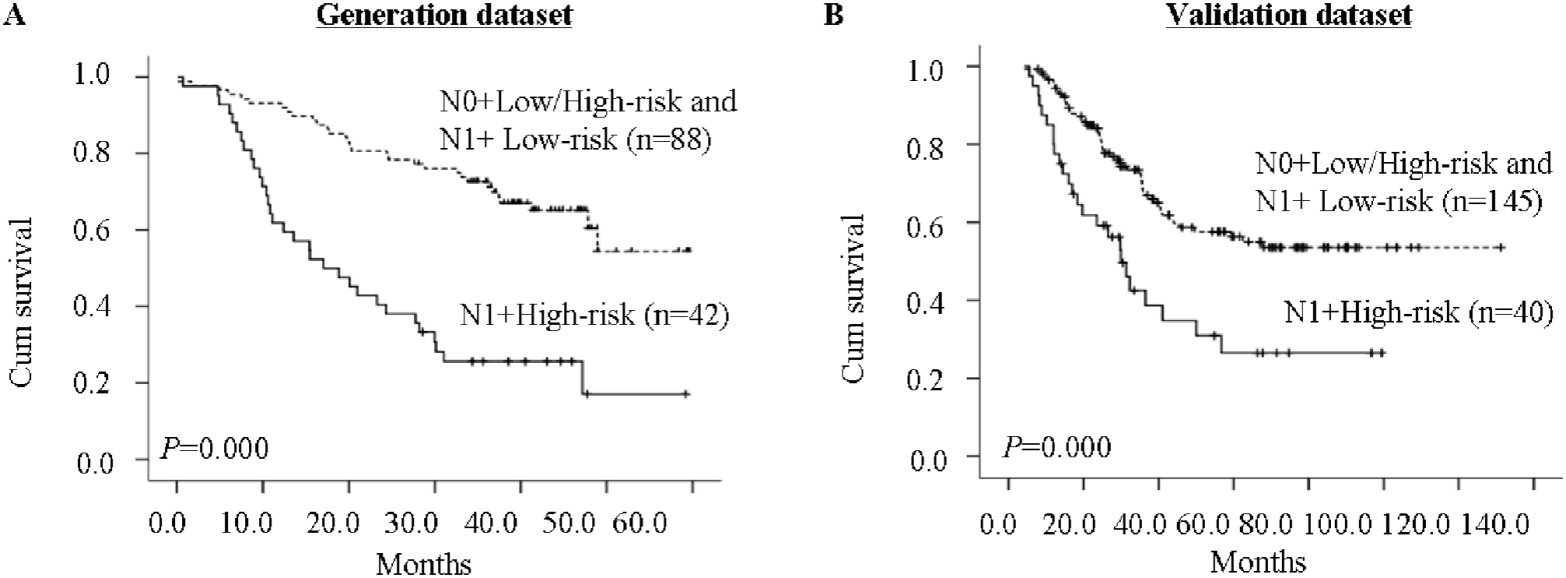
Kaplan-Meier analyses of OS considering a molecular prognostic model and N-stage in generation and validation datasets.

As our results indicate that both the prognostic model and N-stage are involved in ESCC prognosis, we next considered these characteristics together. Patients were subdivided into four subgroups: N0+low-risk, N0+high-risk, N1+low-risk, and N1+high-risk. N1+high-risk patients had the poorest prognoses, whereas the other three groups showed no notable differences in prognoses (data not shown); therefore, these three groups were merged into a single group. Kaplan-Meier curves showed significant differences in OS between the two groups (Figure 3). In the generation dataset, the 3-year OS was 25.6% for the N1+high-risk group compared with 72.7% for the other group. In the validation dataset, the 3- and 5-year OS were 42.5% and 26.5%, respectively, for the N1+high-risk group, compared with 67.9% and 56.3% for the other group.

## Discussion

Although many prospective studies have assessed potential biomarkers of cancer using high-throughput screening techniques [21]–[23], there is often little to no biological connection among the individual biomarkers. Furthermore, single biomarker predictor models often have limited power to predict cancer patient survival [26]–[28]. Therefore, the three-gene signature discovered in the present study, which is comprised of molecules within the EGFR/ERK/Fascin signaling pathway, may represent a useful preclinical model for improving ESCC treatment and clinical outcome. Using two independent cohorts of ESCC patients, our study both generates and validates this molecular prognostic model, which predicts poor prognosis. We investigated our molecular prognostic model at the protein level for two reasons. First, formalin-fixed, paraffin-embedded tissue is far more available than other types of samples such as fresh-frozen tissue. Second, IHC is technically simple, fast, economical, clinical applicable, and robust, in contrast to assessments of gene expression at the mRNA level, which require standardization of techniques to allow comparison of data across days, laboratories, and types of samples [3].

This prognostic model made it possible to identify a cohort of ESCC patients with a 5-year survival of 52%, which is remarkable for this disease. Combining the prognostic model with N-stage, we found that N1+high-risk patients had the poorest clinical outcome, whereas N1+low-risk and N0+high/low-risk patients had similar prognoses. This result, while surprising, could serve to guide treatment options. That is, N1 and high-risk patients may urgently require therapeutic intervention to improve their prognosis. EGFR is a particularly promising molecular target of therapy, as EGFR inhibitors have been widely applied to a variety of solid tumors, such as lung cancer [13], [14], colorectal cancer [29], breast cancer [30], and even ESCC [31]. Some of these therapeutic strategies have been subject to clinical trials, with four EGFR inhibitors currently approved by the US Food and Drug Administration, including gefitinib, erlotinib, cetuximab, and, most recently, panitumumab. Therefore, the poor clinical outcome of N1+high-risk patients might be improved by a more comprehensive treatment approach, such as chemotherapy or radiotherapy combined with cetuximab treatment. In addition to EGFR, Fascin is also recognized as a therapeutic target [32], as binding with migrastatin analogues inhibits Fascin activity and blocks tumor metastasis [33]. Our prognostic model could therefore lead to new avenues of therapy for patients with ESCC, such as treatment with EGFR and/or Fascin inhibitors.

Besides, in the past, patients once diagnosed with lymphatic metastasis received several simultaneous treatments in an unselective manner. However, such overtreatment often fails to improve prognosis and leads to a massive waste of medical resources. Our results also suggest that N1+low-risk patients could be treated the same as lymphonodus-negative patients. Therefore, this new prognostic stratification could guide treatment choices for patients diagnosed with lymphatic metastasis. As exploring potential biomarkers within a single signaling pathway may still be rather limited, future studies could attempt to evaluate multiple signaling pathways to further elucidate the pathogenesis of ESCC in a deeper and more biologically relevant context. Furthermore, we envision that it will be possible to combine other clinical characteristics, such as pTNM-stage, with multi-molecular prognostic model to more accurately predict ESCC survival.

In conclusion, we found that a molecular prognostic model, comprised of expression of EGFR, p-Sp1, and Fascin proteins, was significantly associated with poor ESCC clinical outcome. Such poor survival of patients could be improved by combining chemoradiation with targeted anti-EGFR or/and anti-Fascin treatment. These findings could give rise to a new prognostic stratification system and provide a useful framework for future work on predictive molecular signatures and therapeutic options for ESCC.

## Supporting Information

Figure S1 **Representative images of IHC staining for EGFR, p-Sp1, and Fascin in epithelium tissue adjacent to carcinoma.** Scale bars = 50 µm.
(PDF)

Figure S2 **Correlation analyses between three proteins expression (EGFR, p-Sp1 and Fascin) in generation dataset of 130 cases (A, B and C) and validation dataset of 185 cases (D, E and F).**
(PDF)

Figure S3 **Kaplan-Meier analysis of overall survival for EGFR, p-Sp1 and Fascin in generation dataset of 130 cases (A, B and C) and validation dataset of 185 cases (D, E and F).**
(PDF)

Figure S4 **The predictive ability of the molecular prognostic model compared with individual markers and other clinical prognostic parameters by receiver operating characteristic (ROC) curves (A for generation dataset, B for validation dataset).** The areas under the curve (AUCs) with 95% CI for OS are shown in C (generation dataset) and D (validation dataset).
(PDF)

## References

[1] Oesophageal cancer statistics website. Available: http://www.cancerresearchuk.org/cancer-info/cancerstats/types/oesophagus/. Updated: 2014 April 17.

[2] Li LW, Li YY, Li XY, Zhang CP, Zhou Y, et al (2011) A novel tumor suppressor gene ECRG4 interacts directly with TMPRSS11A (ECRG1) to inhibit cancer cell growth in esophageal carcinoma. BMC Cancer 11: 52–59. 21288367 10.1186/1471-2407-11-52 PMC3039630

[3] Peters CJ, Rees JR, Hardwick RH, Hardwick JS, Vowler SL, et al (2010) A 4-gene signature predicts survival of patients with resected adenocarcinoma of the esophagus, junction, and gastric cardia. Gastroenterology 139: 1995–2004. 20621683 10.1053/j.gastro.2010.05.080

[4] Benowitz S (2008) Revised guidelines signal that gene expression profiles are coming of age. J Natl Cancer Inst 100: 916–917. 18577742 10.1093/jnci/djn228

[5] Ludwig JA, Weinstein JN (2005) Biomarkers in cancer staging, prognosis and treatment selection. Nat Rev Cancer 5: 845–856. 16239904 10.1038/nrc1739

[6] van’t Veer LJ, Dai H, van de Vijver MJ, He YD, Hart AA, et al (2002) Gene expression profiling predicts clinical outcome of breast cancer. Nature 415: 530–536. 11823860 10.1038/415530a

[7] Paik S, Shak S, Tang G, Kim C, Baker J, et al (2004) A multigene assay to predict recurrence of tamoxifen-treated, node-negative breast cancer. N Engl J Med 351: 2817–2826. 15591335 10.1056/NEJMoa041588

[8] Sveen A, Ågesen TH, Nesbakken A, Meling GI, Rognum TO, et al (2012) ColoGuidePro: A Prognostic 7-Gene Expression Signature for Stage III Colorectal Cancer Patients. Clin Cancer Res 18: 6001–6010. 22991413 10.1158/1078-0432.CCR-11-3302

[9] Hoffmann AC, Danenberg KD, Taubert H, Danenberg PV, Wuerl P (2009) A Three-Gene Signature for Outcome in Soft Tissue Sarcoma. Clin Cancer Res 15: 5191–5198. 19671876 10.1158/1078-0432.CCR-08-2534

[10] Lu XF, Li EM, Du ZP, Xie JJ, Guo ZY, et al (2010) Specificity protein 1 regulates fascin expression in esophageal squamous cell carcinoma as the result of the epidermal growth factor/extracellular signal-regulated kinase signaling pathway activation. Cell Mol Life Sci 67: 3313–3329. 20502940 10.1007/s00018-010-0382-y PMC11115853

[11] Nicholson RI, Gee JM, Harper ME (2001) EGFR and cancer prognosis. Eur J Cancer 37 Suppl 4S9–15. 11597399 10.1016/s0959-8049(01)00231-3

[12] Anonymous (2007) Targeting HER1/EGFR: Pathway to Progress in Cancer Therapy. Oncology (Williston Park) 21: 2 p preceding 1754. 18247019

[13] Dutta PR, Maity A (2007) Cellular responses to EGFR inhibitors and their relevance to cancer therapy. Cancer Lett 254: 165–177. 17367921 10.1016/j.canlet.2007.02.006 PMC1986742

[14] Seshacharyulu P, Ponnusamy MP, Haridas D, Jain M, Ganti AK, et al (2012) Targeting the EGFR signaling pathway in cancer therapy. Expert Opin Ther Targets 16: 15–31. 22239438 10.1517/14728222.2011.648617 PMC3291787

[15] Grothey A, Hashizume R, Sahin AA, McCrea PD (2000) Fascin, an actin-bundling protein associated with cell motility, is upregulated in hormone receptor negative breast cancer. Br J Cancer 83: 870–873. 10970687 10.1054/bjoc.2000.1395 PMC2374674

[16] Hashimoto Y, Ito T, Inoue H, Okumura T, Tanaka E, et al (2005) Prognostic significance of fascin overexpression in human esophageal squamous cell carcinoma. Clin Cancer Res 11: 2597–2605. 15814639 10.1158/1078-0432.CCR-04-1378

[17] Zhao Q, Shen JH, Shen ZY, Wu ZY, Xu XE, et al (2010) Phosphorylation of Fascin Decreases the Risk of Poor Survival in Patients With Esophageal Squamous Cell Carcinoma. J Histochem Cytochem 58: 979–988. 20713986 10.1369/jhc.2010.955765 PMC2958140

[18] Sun LL, Sun XX, Xu XE, Zhu MX, Wu ZY, et al (2013) Overexpression of Jumonji AT-rich interactive domain 1B and PHD finger protein 2 is involved in the progression of esophageal squamous cell carcinoma. Acta Histochem 115: 56–62. 22534467 10.1016/j.acthis.2012.04.001

[19] Xu QX, Li EM, Zhang YF, Liao LD, Xu XE, et al (2012) Overexpression of sigma1 receptor and its positive associations with pathologic TNM classification in esophageal squamous cell carcinoma. J Histochem Cytochem 60: 457–466. 22511599 10.1369/0022155412443542 PMC3393071

[20] Bilous M, Dowsett M, Hanna W, Isola J, Lebeau A, et al (2003) Current perspectives on HER2 testing: a review of National Testing Guidelines. Mod Pathol 16: 173–182. 12591971 10.1097/01.MP.0000052102.90815.82

[21] Yang XR, Xu Y, Yu B, Zhou J, Qiu SJ, et al (2010) High expression levels of putative hepatic stem/progenitor cell biomarkers related to tumour angiogenesis and poor prognosis of hepatocellular carcinoma. Gut 59: 953–962. 20442200 10.1136/gut.2008.176271

[22] Kadara H, Behrens C, Yuan P, Solis L, Liu D, et al (2011) A Five-Gene and Corresponding Protein Signature for Stage-I Lung Adenocarcinoma Prognosis. Clin Cancer Res 17: 1490–1501. 21163870 10.1158/1078-0432.CCR-10-2703 PMC3079395

[23] Garcia I, Mayol G, Ríos J, Domenech G, Cheung NK, et al (2012) A Three-Gene Expression Signature Model for Risk Stratification of Patients with Neuroblastoma. Clin Cancer Res 18: 2012–2023. 22328561 10.1158/1078-0432.CCR-11-2483 PMC4240975

[24] Gonzaga IM, Soares-Lima SC, de Santos PT, Blanco TC, et al (2012) Alterations in epidermal growth factor receptors 1 and 2 in esophageal squamous cell carcinomas. BMC Cancer 12: 569–579. 23207070 10.1186/1471-2407-12-569 PMC3537527

[25] Zhang H, Xu L, Xiao D, Xie J, Zeng H, et al (2006) Fascin is a potential biomarker for early-stage oesophageal squamous cell carcinoma. J Clin Pathol 59: 958–964. 16524962 10.1136/jcp.2005.032730 PMC1860492

[26] Krikelis D, Bobos M, Karayannopoulou G, Resiga L, Chrysafi S, et al (2013) Expression profiling of 21 biomolecules in locally advanced nasopharyngeal carcinomas of Caucasian patients. BMC Clin Pathol 13: 1–15. 23360534 10.1186/1472-6890-13-1 PMC3563444

[27] Zhu H, Bhaijee F, Ishaq N, Pepper DJ, Backus K, et al (2013) Correlation of Notch1, pAKT and nuclear NF-κB expression in triple negative breast cancer. Am J Cancer Res 3: 230–239. 23593544 PMC3623841

[28] Gou HF, Li X, Qiu M, Cheng K, Li LH, et al (2013) Epidermal growth factor receptor (EGFR)-RAS signaling pathway in penile squamous cell carcinoma. PLoS One 8: e62175–62181. 23637996 10.1371/journal.pone.0062175 PMC3634795

[29] Heinemann V, Stintzing S, Kirchner T, Boeck S, Jung A (2009) Clinical relevance of EGFR- and KRAS-status in colorectal cancer patients treated with monoclonal antibodies directed against the EGFR. Cancer Treat Rev 35: 262–271. 19117687 10.1016/j.ctrv.2008.11.005

[30] Carey LA, Rugo HS, Marcom PK, Mayer EL, Esteva FJ, et al (2012) TBCRC 001: randomized phase II study of cetuximab in combination with carboplatin in stage IV triple-negativebreast cancer. J Clin Oncol 30: 2615–2623. 22665533 10.1200/JCO.2010.34.5579 PMC3413275

[31] Chen Y, Wu X, Bu S, He C, Wang W, et al (2012) Promising outcomes of definitive chemoradiation and cetuximab for patients with esophageal squamous cell carcinoma. Cancer Sci 103: 1979–1984. 22845557 10.1111/j.1349-7006.2012.02393.x PMC5439109

[32] Tan VY, Lewis SJ, Adams JC, Martin RM (2013) Association of fascin-1 with mortality, disease progression and metastasis in carcinomas: a systematic review and meta-analysis. BMC Med 11: 52–68. 23442983 10.1186/1741-7015-11-52 PMC3635876

[33] Chen L, Yang S, Jakoncic J, Zhang JJ, Huang XY (2010) Migrastatin analogues target fascin to block tumour metastasis. Nature 464: 1062–1066. 20393565 10.1038/nature08978 PMC2857318

