# Supplementary material for: A Molecular Prognostic Model Predicts Esophageal Squamous Cell Carcinoma Prognosis
Source: PLoS One. 2014 Aug 25;9(8):e106007. doi: 10.1371/journal.pone.0106007 (PMC4143329; doi:10.1371/journal.pone.0106007)
Supplement: Figure S1 — Representative images of IHC staining for EGFR, p-Sp1, and Fascin in epithelium tissue adjacent to carcinoma. Scale bars = 50 µm. (PDF) [file pone.0106007.s001.pdf]

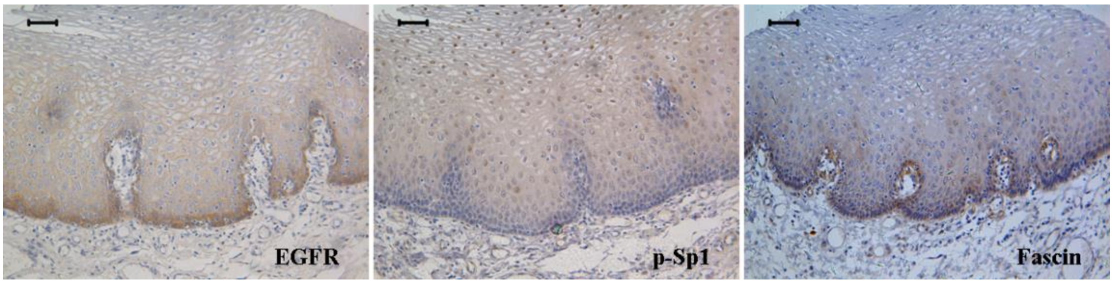

Figure S1 Representative images of IHC staining for EGFR, p-Sp1, and Fascin in epithelium tissue adjacent to carcinoma. Scale bars = 50 μm.
