# Supplementary material for: A Molecular Prognostic Model Predicts Esophageal Squamous Cell Carcinoma Prognosis
Source: PLoS One. 2014 Aug 25;9(8):e106007. doi: 10.1371/journal.pone.0106007 (PMC4143329; doi:10.1371/journal.pone.0106007)
Supplement: Figure S3 — Kaplan-Meier analysis of overall survival for EGFR, p-Sp1 and Fascin in generation dataset of 130 cases (A, B and C) and validation dataset of 185 cases (D, E and F). (PDF) [file pone.0106007.s003.pdf]

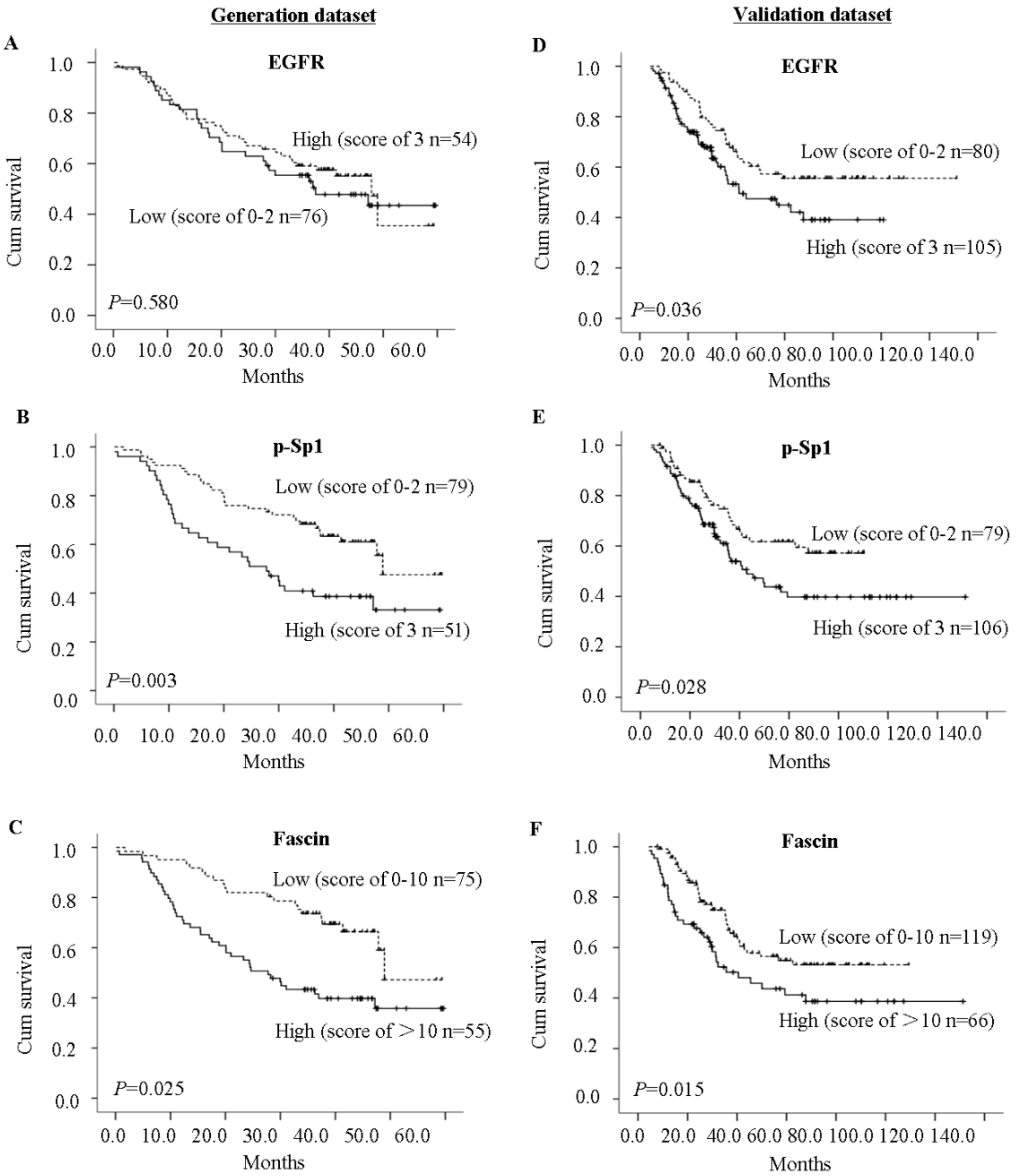

Figure S3 Kaplan-Meier analysis of overall survival for EGFR, p-Sp1 and Fascin in generation dataset of 130 cases (A, B and C) and validation dataset of 185 cases (D, E and F).
