# Supplementary material for: A Molecular Prognostic Model Predicts Esophageal Squamous Cell Carcinoma Prognosis
Source: PLoS One. 2014 Aug 25;9(8):e106007. doi: 10.1371/journal.pone.0106007 (PMC4143329; doi:10.1371/journal.pone.0106007)
Supplement: Figure S4 — The predictive ability of the molecular prognostic model compared with individual markers and other clinical prognostic parameters by receiver operating characteristic (ROC) curves (A for generation dataset, B for validation dataset). The areas under the curve (AUCs) with 95% CI for OS are shown in C (generation dataset) and D (validation dataset). (PDF) [file pone.0106007.s004.pdf]

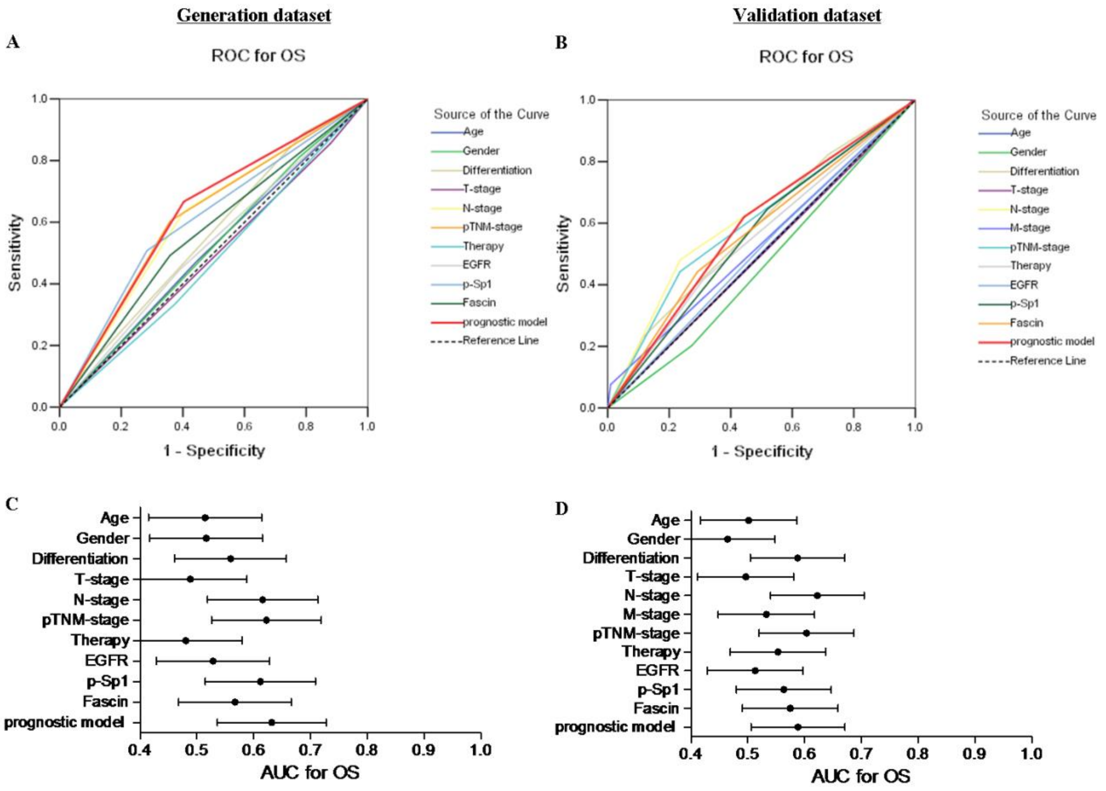

Figure S4 The predictive ability of the molecular prognostic model compared with individual markers and other clinical prognostic parameters by receiver operating characteristic (ROC) curves (A for generation dataset, B for validation dataset). The areas under the curve (AUCs) with 95% CI for OS are shown in C (generation dataset) and D (validation dataset).
